# Supraspinous ligament arc tangent guided freehand thoracic pedicle screw insertion technique: high parallelism between screws and upper endplate [Author-notes fns01]

**DOI:** 10.3389/fsurg.2023.1219816

**Published:** 2023-08-07

**Authors:** Fushuai Peng, Meng Gao, Qiang Li, Zhensong Jiang, Fei Chen, Mingtong Sun, Yudong Lai, Haoyu Wang, Xingpeng Wang, Tao Li, Wen Zhang

**Affiliations:** ^1^ Department of Spine Surgery, Shandong Provincial Hospital Affiliated to Shandong First Medical University, Jinan, China; ^2^ Department of Radiology, The Second Affiliated Hospital of Shandong First Medical University, Taian, China

**Keywords:** freehand technique, Supraspinous ligament Arc Tangent, thoracic pedicle, screws, parallelism

## Abstract

**Research objective:**

To propose a technique for placing pedicle screws in the thoracic spine using the Supraspinous ligament Arc Tangent (SLAT) as a guide to increase the safety and stability of screw placement.

**Content and methods:**

A retrospective analysis of postoperative anteroposterior and lateral x-ray images was performed for 118 patients with thoracic spine diseases who received conventional freehand technique from January 2016 to May 2020 and SLAT-guided technique since June 2020 to present. The diagnoses included thoracic spinal stenosis, deformity, fractures, infections, and tumors. The angle between the screw and the upper endplate was categorized as grade 1 (0°–5°), grade 2 (5°–10°), and grade 3 (>10°). Three surgeons with more than 10 years of experience in spinal surgery measured the angle between the screw and the upper endplate in the lateral view. Chi-square test was used for statistical analysis, and *p* < 0.05 was considered statistically significant.

**Results:**

A total of 1315 pedicle screws were placed from T1 to T12 in all patients. In the conventional freehand technique group, 549 screws were grade 1, 35 screws were grade 2, and 23 screws were grade 3. In the SLAT-guided freehand technique group, 685 screws were grade 1, 15 screws were grade 2, and 8 screws were grade 3. The data of each group was *p* < 0.05 by Chi-squared test, which was statistically significant, indicating that the SLAT-guided freehand technique resulted in a higher rate of parallelism between the screws and the upper endplate. All patients underwent intraoperative neurophysiological monitoring, immediate postoperative neurological examination, postoperative x-ray examination, and assess the eventual recovery. The screws were safe and stable, and no complications related to pedicle screw placement were found.

**Conclusion:**

The SLAT-guided freehand technique for placing pedicle screws in the thoracic spine can achieve a higher rate of screw-upper endplate parallelism, making screw placement safer and more accurate. Our method provides a convenient and reliable technique for most spinal surgeons, allowing for increased accuracy and safety with less fluoroscopic guidance.

## 1. Introduction

Since the description of the pedicle screw-rod system in 1963 by Roy-Camille, this technique has been constantly developed and has become increasingly mature, and is now one of the commonly used techniques by spine surgeons (1, 2). Because this technique can fix the three columns of the spine and has strong three-dimensional corrective ability, it is often used to treat diseases such as spinal degeneration, deformity, fractures, infections, and tumors (1, 3–10). This technique was first applied to the lumbar spine (1), Subsequently, some scholars proposed that this technique could also be used to treat thoracic spine diseases. However, human-specific anatomical structures show that the pedicles of the thoracic spine are thinner than those of the lumbar spine (11–13), and the screws placed close to the vertebral canal, blood vessels, and important organs may pose potential risks, even though this technique has been continuously improved in recent years, and there are related literature reports that this technique can safely and reliably treat all types of diseases in the thoracic spine (13, 14). Inappropriate screw placement may cause irreversible complications. According to relevant literature reports, there is a certain correlation between improper screw placement and screw loosening, screw fracture, pedicle fracture, dura mater tearing, and poor reduction (15–19). Therefore, accurate and safe pedicle screw placement methods are of great significance.

Various safe methods for pedicle screw placement have been reported, including freehand pedicle screw placement techniques (13, 20, 21), fluoroscopy-guided techniques (22, 23), optoelectronic navigation technology (24), 3D printing personalized navigation technology (25, 26), and robot-guided technology (27, 28). Each method has its own advantages and disadvantages. The classic pedicle screw placement technique guided by fluoroscopy has significant advantages in terms of accuracy and safety, but it exposes both doctors and patients to more radiation. Emerging technologies such as photonic navigation, 3D-printed personalized navigation, and robotic guidance techniques reduce additional radiation exposure, but their practicality and popularity have not yet reached satisfactory levels. In contrast, freehand pedicle screw placement techniques have a series of advantages, such as reducing intraoperative radiation exposure, shortening surgery time, and reducing patients' economic burden (14, 29–31), but this technique heavily relies on the surgeon's experience and has a long learning curve for beginners. Furthermore, this technique does not allow surgeons to observe the movement of the screw in real-time, which creates a certain hidden danger. Therefore, a simple and safe freehand pedicle screw placement technique is particularly important.

**Figure 1:**
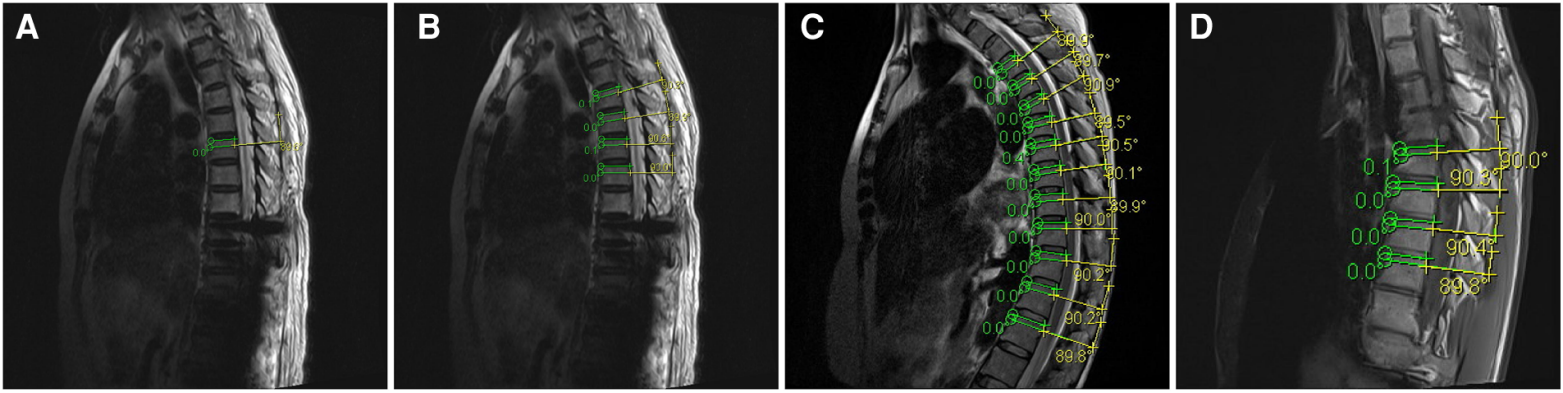
(**A**) Phenomenon of the supraspinous ligament arch tangent being perpendicular to the endplate of the corresponding vertebra on MRI; (**B**) schematic diagram of preoperative MRI measurement of a patient with thoracic spinal stenosis; (**C**) schematic diagram of preoperative MRI measurement of a patient with spinal kyphosis; (**D**) schematic diagram of preoperative MRI measurement of a patient with spinal scoliosis.

The anatomical structure of the thoracic spine demonstrates that the supraspinous ligament is relatively stable and does not have the same degree of mobility as the lumbar spine. Therefore, it can serve as a reference landmark during surgery. During our study, we found that the arc tangent of the supraspinous ligament in the thoracic spine is perpendicular to the superior endplate of the corresponding vertebra (Figure 1A). We randomly selected preoperative MRI images of 10 patients, including those with scoliosis, kyphosis, and without any deformities, and measured the angle between the supraspinous ligament arc tangent and the superior endplate on the images, which verified the good perpendicular relationship between the two (Figures 1B–D). Based on the above two aspects, we propose a freehand pedicle screw placement technique guided by the supraspinous ligament arc tangent (SLAT) for the thoracic spine, aiming to safely implant screws parallel to the superior endplate and achieve stable spinal fixation with a rod.

## 2. Content and methods

### 2.1. Inclusion criteria

This retrospective study reviewed the data of 58 patients with thoracic spine diseases treated with conventional freehand techniques by senior spinal surgeons in our department from January 2016 to May 2020, and 60 patients with thoracic spine diseases treated with SLAT-guided freehand techniques from June 2020 to present (Table 1). The diagnoses included: (1) spinal scoliosis (including adolescent idiopathic scoliosis, congenital scoliosis, and neurofibromatosis-associated scoliosis, etc.); (2) spinal kyphosis (including Scheuermann's disease, ankylosing spondylitis-associated kyphosis, etc.); (3) common diseases (including thoracic spinal stenosis, spinal fractures, infections, and tumors, etc.). This study was approved by the Ethics Committee of the Provincial Hospital affiliated with Shandong First Medical University and informed consent was obtained from each patient or their guardian.

**Table 1:** Patient statistics.

Disease category	Group	
	Conventional freehand technique group (Number of patients)	SLAT-guided freehand technique group (Number of patients)
Spinal scoliosis	20	19
Spinal kyphosis	11	23
Thoracic spinal stenosis	16	10
Spinal fractures	7	5
Infections	1	1
Tumors	3	2
Total	58	60

### 2.2. SLAT guided freehand technique

**Figure 2:**
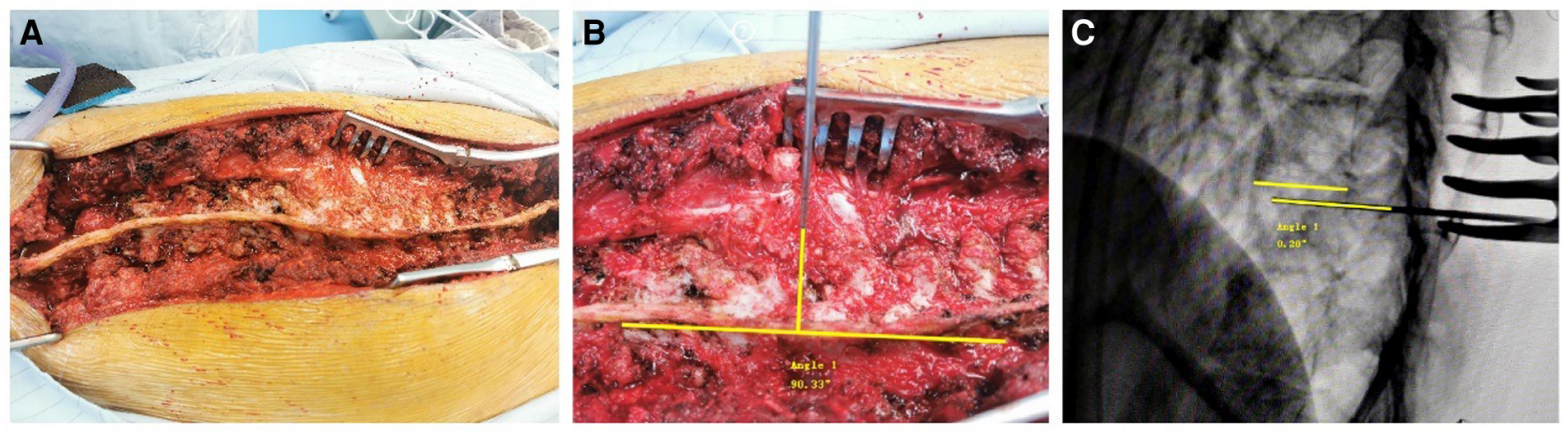
(**A**) Schematic diagram of accurate exposure of the supraspinous ligament in a patient with spinal scoliosis during surgery; (**B**) schematic diagram of the insertion of a straight probe perpendicular to the tangent of the supraspinous ligament arch; (**C**) schematic diagram of a probe almost horizontally aligned with the upper endplate under fluoroscopy.

After adequate preoperative preparation, the patient who had undergone general anesthesia was placed prone on the operating table. The area that needs to be operated on is identified by pressing the spinous process and using fluoroscopy, and then the skin is incised along the midline of the spinous process. The subcutaneous fat, muscles, and fascia are dissected using an electric scalpel, exposing the supraspinous ligament, facet joints, lamina, and transverse process. At the junction of the upper articular process and transverse process, an opening is made at a position 3 mm from the tail. A probe is used to detect the length from the pedicle of the vertebral arch to the anterior edge of the vertebral body. The horizontal direction is inclined inward by 30° at T1 and T2 levels, and inward by 20° at T3 to T12 levels. Subsequently, the screw channel is expanded using a wire tap perpendicular to the arc tangent of the corresponding spinal supraspinal ligament (Figures 2A–C). The pedicle wall is detected with a soft probe, and after confirming that there is no breach of the pedicle wall, the corresponding screw is placed into the prepared channel. After all screws are placed, a connecting rod is inserted, and lateral fluoroscopy is performed using a C-arm. Intraoperative neurophysiological monitoring was used for all patients, and early neurological examination was performed immediately after the surgery to adjust the screws suspected to be misplaced.

### 2.3. Measuring methods

**Figure 3:**
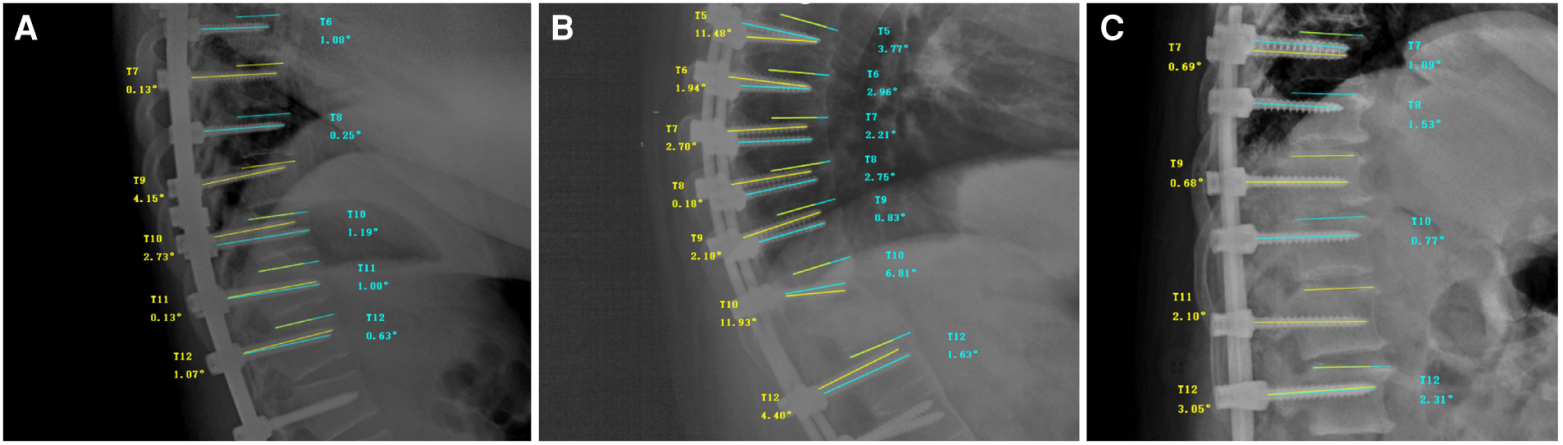
(**A**) Schematic diagram of postoperative measurement in a patient with spinal scoliosis; (**B**) schematic diagram of postoperative measurement in a patient with spinal kyphosis; (**C**) schematic diagram of postoperative measurement in a patient with thoracic spinal stenosis.

We identified whether the screws were placed on the left or right side in the anteroposterior radiographs, and measured the angle between the screws and the upper endplate in the lateral radiographs (Figures 3A–C). All postoperative images were measured by three surgeons with more than 10 years of spinal surgery experience, and the angle between the screw and the upper endplate was classified as grade 1 for 0° to 5°, grade 2 for 5° to 10°, and grade 3 for over 10°. All the measurement data were collected and the final results were summarized, and there was no statistical difference among the measurers. All patients underwent intraoperative fluoroscopy and neurophysiological monitoring, and early neurological examination was performed immediately after the operation. Any suspected improperly placed screws were adjusted in a timely manner.

### 2.4. Statistical analysis

SPSS 13.0 software (SPSS Inc., Chicago, Illinois, USA) was used for statistical analysis. Chi-square test was performed on the collected data. In the analysis, *p* < 0.05 indicates statistical significance. There was no difference between the measurers.

## 3. Results

A total of 1,315 pedicle screws were included during the study period. There were 2 (0.15%) screws at T1, 23 (1.75%) screws at T2, 42 (3.19%) screws at T3, 79 (6.01%) screws at T4, 124 (9.43%) screws at T5, 137 (10.42%) screws at T6, 137 (10.42%) screws at T7, 147 (11.18%) screws at T8, 159 (12.09%) screws at T9, 146 (11.10%) screws at T10, 163 (12.40%) screws at T11, and 156 (11.86%) screws at T12. The angles between the pedicle screws and the upper endplate at each level were measured by three surgeons. Using conventional freehand technique, A total of 293 screws were placed in 20 patients with spinal scoliosis, 176 screws were placed in 11 patients with spinal kyphosis, and 138 screws were placed in 27 patients with common diseases (16 with thoracic spinal stenosis, 7 with spinal fractures, 1 with infection, and 3 with tumors). Using SLAT-guided freehand technique, A total of 254 screws were placed in 19 patients with spinal scoliosis, 342 screws were placed in 23 patients with spinal kyphosis, and 112 screws were placed in 18 patients with common diseases (10 with thoracic spinal stenosis, 5 with spinal fractures, 1 with infection, and 2 with tumors). In the conventional freehand technique group, The number of grade 1 screws was 549 (90.44%), 35 (5.77%) were grade 2, and 23 (3.79%) were grade 3; In the SLAT-guided freehand technique group, The number of grade 1 screws was 685 (96.75%), 15(2.12%) were grade 2, and 8 (1.13%) were grade 3 (Table 2). No abnormally placed screws were found during the operation, and no complications related to pedicle screws were found during hospitalization. After processing and analyzing the data, the results of Chi-squared test showed that *p* ≈ 0.037 < 0.05 in scoliosis group, *p* ≈ 0.046 < 0.05 in kyphosis group, *p* ≈ 0.000 < 0.05 in conventional disease group and *p* ≈ 0.000 < 0.05 in total, and the first level rate of SLAT group was higher than that of the conventional technology group, indicating that the SLAT-guided freehand technique resulted in a higher rate of parallelism between the screws and the upper endplate. All patients underwent intraoperative neurophysiological monitoring, immediate postoperative neurological examination, postoperative x-ray examination, and assess the eventual recovery (Table 3). The screws were safe and stable, and no complications related to pedicle screw placement were found. In order to reduce the number of times patients receive radiation, we will no longer require patients to undergo routine CT examinations after evaluating their postoperative effectiveness.

**Table 2:** Number of screws of different grade and levels of thoracic vertebrae in different groups and techniques (Conventional freehand technology group replaced by A; SLAT-guided freehand technique group replaced by B).

Thoracic vertebra	Group					
	Scoliosis group		Kyphosis group		Conventional diseases group	
	A	B	A	B	A	B
	Grade 1/ Grade 2/ Grade 3		Grade 1/ Grade 2/ Grade 3		Grade 1/ Grade 2/ Grade 3	
T1	0/0/0	0/0/0	0/0/0	2/0/0	0/0/0	0/0/0
T2	6/0/1	4/0/0	6/2/0	4/0/0	0/0/0	0/0/0
T3	12/2/1	7/1/0	8/0/0	6/2/1	0/0/0	2/0/0
T4	12/2/5	13/0/1	10/1/3	16/2/2	6/2/0	4/0/0
T5	27/2/0	18/1/0	17/1/1	22/1/0	16/2/0	15/0/1
T6	31/1/0	20/0/0	18/2/0	27/1/0	18/0/1	18/0/0
T7	25/1/0	19/1/0	14/1/0	36/0/0	16/3/1	18/2/0
T8	28/0/1	24/0/0	16/0/1	33/0/1	22/2/0	19/0/0
T9	31/1/0	33/1/0	16/1/0	40/0/0	16/2/2	16/0/0
T10	33/0/0	30/0/0	19/0/0	46/0/0	10/0/0	8/0/0
T11	34/1/0	43/0/0	18/0/0	50/1/0	8/1/2	5/0/0
T12	32/2/2	36/1/1	18/2/1	47/1/1	6/1/1	4/0/0
Total	271/12/10	247/5/2	160/10/6	329/8/5	118/13/7	109/2/1

**Table 3:** Comparison of two techniques within different groups and comparison of the sum of three groups. (Conventional freehand technology group replaced by A; SLAT-guided freehand technique group replaced by B).

Group		Grade			*p*
		Grade 1	Grade 2	Grade 3	
Scoliosis	A	271 (92.49%)	12 (4.10%)	10 (3.41%)	0.037
	B	247 (97.24%)	5 (1.97%)	2 (0.79%)	
Kyphosis	A	160 (90.91%)	10 (5.68%)	6 (3.41%)	0.046
	B	329 (96.20%)	8 (2.34%)	5 (1.46%)	
Conventional diseases	A	118 (85.51%)	13 (9.42%)	7 (5.07%)	0.000
	B	109 (97.32%)	2 (1.79%)	1 (0.89%)	
Total	A	549 (90.44%)	35 (5.77%)	23 (3.79%)	0.000
	B	685 (96.75%)	15 (2.12%)	8 (1.13%)	

## 4. Discussion

In the past, there have been many types of freehand techniques for inserting pedicle screws in the thoracic spine, and it has been difficult to standardize the trajectory of screws in the sagittal plane. Although Kim et al. reported an effective freehand technique in 2004, they did not provide a precise sagittal plane trajectory standard (32). In 2008, Karapinar et al., in 2009, Modi H et al., and in 2010, Modi HN et al., all reported their own views, but still did not propose a method for sagittal plane trajectory (33–35). In 2011, Parker, SL et al. supported the view that screws should be parallel to the upper endplate, but achieving this trajectory requires the use of fluoroscopy during the operation (29). In 2014, Fennell, VS et al. and in 2016, Avila, MJ et al.both supported the view that screws should always be orthogonal to the corresponding horizontal physiological curvature of the spine to achieve the parallelism between the screw and the upper endplate, However, this method was later considered difficult to master by Swaminathan, G et al. (13, 36, 37). In 2015, Li, J et al. described a manual technique that referenced the supraspinous ligament, but this study only involved fractures and did not verify the method in scoliosis and kyphosis, making the sample population limited (38). In 2018, Gokcen, HB et al. and in 2019, Zhang, ZF et al. both described a sagittal plane trajectory perpendicular to the vertebral body, but this reference marker is easily affected by the angle of view (21, 39). In 2018, Kim, TH et al. described the direction of the screw perpendicular to the superior articular process, which allows the screw to enter the vertebral body obliquely. However, for vertebrae with thinner pedicles, the usefulness of this method still needs to be further verified (40).

Most scholars support the sagittal plane state in which the screw is parallel to the upper endplate. In 2020, Swaminathan, G et al. verified the Fennell technique through simulation experiments with pedicle screws and proved that inserting screws parallel to the upper endplate is safe (36). In 2021, Jarvers, JS et al. experimentally proved that inserting pedicle screws parallel to the upper endplate in osteoporotic lumbar vertebrae has the best fatigue performance and the maximum resistance to pullout force (41). Therefore, different experiments also support the sagittal plane state where the screw is parallel to the upper endplate. Based on stability and safety, we also agree with the sagittal plane trajectory where the screw is parallel to the upper endplate. However, no one has provided a good reference standard for how to make the screw parallel to the upper endplate. In order to reduce the number of times patients receive radiation, we will no longer require patients to undergo routine CT examinations after evaluating the effectiveness of the surgery, and there is relevant literature indicating that postoperative CT is not necessary either (42).

We propose a simple and standardized technique for the placement of thoracic pedicle screws using only manual techniques. In the sagittal plane, the screw is inserted perpendicular to the arc tangent of the corresponding supraspinous ligament. Our results show that compared to the conventional freehand technique, the use of the arc tangent of the supraspinous ligament as a reference leads to a higher satisfaction rate for parallelism of the screw with the superior endplate. Our technique allows spine surgeons to accurately and easily place pedicle screws, resulting in a safe and stable spinal screw system that maintains parallelism with the superior endplate in the sagittal plane. We not only evaluated the effectiveness of this technique for common diseases, but also further evaluated its effect on scoliosis and kyphosis. We included patients with scoliosis and kyphosis, and achieved satisfactory results, demonstrating that this technique can still be applied when correcting certain types of deformities.

The development of this technique depends on rich clinical experience in pedicle screw placement. Beginners need to overcome a learning curve when evaluating the arc tangent of the supraspinous ligament. Currently, this technique only applies to thoracic vertebrae, and research on cervical and lumbar vertebrae will be conducted in the future. Furthermore, due to individual differences, some extreme cases may require more personalized treatment plans.

## 5. Conclusion

The Supraspinous Ligament Arc Tangent (SLAT) freehand technique, which uses the arc tangent of the supraspinous ligament as a guide for perpendicular insertion of the screw in the sagittal plane, yields satisfactory results. It allows spine surgeons to accurately and safely place screws in the thoracic region, and is a simple and easy-to-learn technique that greatly benefits beginners.

## Data Availability

The data analyzed in this study is subject to the following licenses/restrictions: The data used to support the findings of this study are available from the corresponding author upon request. Requests to access these datasets should be directed to TL, litao19812022@163.com.
